# Subclinical Ovine Gammaherpesvirus 2-Related Infections in Free-Ranging Wild Boars (*Sus scrofa*) from Southern Brazil

**DOI:** 10.3390/pathogens13060515

**Published:** 2024-06-18

**Authors:** Selwyn Arlington Headley, Juliana Torres Tomazi Fritzen, Flavia Helena Pereira Silva, Silvio Luis Marsiglio Minarelli, Leandro Meneguelli Biondo, Louise Bach Kmetiuk, Alexander Welker Biondo, Amauri Alcindo Alfieri

**Affiliations:** 1 Laboratory of Animal Pathology, Department of Preventive Veterinary Medicine, Universidade Estadual de Londrina, Londrina, Paraná 86057-970, Brazil; flaviahelena.pereira@uel.br; 2 Multi-User Animal Health Laboratory (LAMSA), Department of Preventive Veterinary Medicine, Universidade Estadual de Londrina, Londrina 86057-970, Brazil; alfieri@uel.br; 3 Laboratory of Animal Virology, Department of Preventive Veterinary Medicine, Universidade Estadual de Londrina, Londrina 86057-970, Brazil; jufritzen@uel.br (J.T.T.F.); silvio.minarelli@uel.br (S.L.M.M.); 4 National Institute of the Atlantic Forest (INMA), Brazilian Ministry of Science, Technology, and Innovation, Santa Teresa 29650-000, Brazil; leandro.biondo@inma.gov.br; 5 Zoonosis Surveillance Unit, City Secretary of Health, Curitiba 81265-320, Brazil; louisebachk@gmail.com; 6 Department of Veterinary Medicine, Federal University of Paraná, Curitiba 80035-050, Brazil; abiondo@ufpr.br

**Keywords:** bridge host, disease transmission, interstitial pneumonia, *Macavirus*, malignant catarrhal fever, molecular epidemiology, 15A antigenic epitope

## Abstract

Ovine gammaherpesvirus 2 (OvGHV2), is a *Macavirus* and the cause of sheep-associated malignant catarrhal fever (SA-MCF), in which sheep are the asymptomatic reservoir hosts. Susceptible mammalian populations infected by OvGHV2 may develop clinical SA-MCF or subclinical infections. All members of the *Macavirus* genus known to be associated with MCF are collectively referred to as the MCF virus (MCFV) complex. This report describes the occurrence of subclinical OvGHV2-related infections in free-ranging wild boars (*Sus scrofa*) from southern Brazil. Specific body organs (*n* = 14) and biological samples (nasal and oral swabs; *n* = 17) were collected from 24 asymptomatic wild boars from a conservation unit located within the Central-eastern mesoregion of Paraná State. Organs were processed to observe histopathological patterns suggestive of diseases of domestic animals; only pulmonary samples were used in an immunohistochemical assay designed to detect MCFV tissue antigens. Furthermore, all samples were submitted to molecular assays designed to detect the OvGHV2 tegument protein gene. Viral-induced pneumonia was diagnosed in two wild boars; one of these contained OvGHV2 DNA, with MCFV antigens identified in the other. Additionally, MCFV tissue antigens were detected within pulmonary epithelial cells of the lungs with and without pulmonary disease. Collectively, OvGHV2 was detected in 37.5% (9/24) of all wild boars, with detection occurring in the organs of 57.1% (8/14) wild boars and the oral cavity of one animal. These results demonstrated that these wild boars were subclinically infected by OvGHV2, and that infection produced typical pulmonary alterations. In addition, the detection of OvGHV2 within the oral cavity of one wild boar may suggest that this animal may be a potential disseminator of this pathogen to susceptible animal populations, including livestock and wildlife, acting as a possible bridge host for OvGHV2. Furthermore, infection by OvGHV2 probably occurred due to incidental contact with asymptomatic sheep maintained within the surrounding rural areas and not within the conservation units.

## 1. Introduction

Ovine gammaherpesvirus 2 (OvGHV2) is an infectious disease pathogen that causes sheep-associated malignant catarrhal fever (SA-MCF) in a wide range of mammalian hosts worldwide [1,2,3,4]. Furthermore, OvGHV2 is a member of the *Macavirus* genus, subfamily *Gammaherpesvirinae* [5], which share the 15A (15A) antigenic epitope with all recognized viral pathogens associated with the development of malignant catarrhal fever (MCF) in specific mammalian hosts [6,7]. These group of viruses are referred to as the MCF virus (MCFV) complex, are antigenically and phylogenetically related [8,9], and are well conserved within the DNA polymerase gene [9]. Other members of the *Macavirus* genus known to be associated with the development of MCF include Alcelaphine gammaherpesvirus 1 and 2, and Caprine gammaherpesvirus 2 [2,3,4]. Additionally, there are several members of the *Macavirus* genus [5] that have not been associated with any specific disease condition in mammalian populations.

The histological hallmark of OvGHV2-induced infections in mammalian hosts is marked proliferation of lymphocytes with disseminated vascular lesions [3,4,10]. Additionally, our research group standardized an immunohistochemical (IHC) assay to detect tissue antigens common to members of the MCFV complex using the 15A-monoclonal antibody, 15A-MAb [11]. Therefore, confirmation of the MCFV tissue antigen detected in animals requires complementary molecular testing to determine the specific *Macavirus* identified by IHC.

Sheep are the asymptomatic reservoir hosts for OvGHV2 with clinical manifestations of SA-MCF occurring in susceptible animal populations [1,2,3]. Experimental studies have confirmed that infections by OvGHV2 in susceptible hosts may occur due to the direct contact with the nasal secretions of asymptomatic sheep [12,13,14], demonstrating that this is most the important epidemiological form of transmission. Nevertheless, transplacental infection of OvGHV2 was confirmed between an infected cow and a fetus [15], and infection via artificial insemination was suspected on a commercial pig farm [16], while OvGHV2 was identified in fetal tissues of cattle [17,18] and in the male reproductive system of sheep [19]. Collectively, these findings suggest the occurrence of vertical infection due to OvGHV2.

Normally, animals infected with OvGHV2 develop the most frequent clinical manifestations of SA-MCF, referred to as the “head and eye” form, which is typically characterized by elevated fever, corneal edema, serous nasal and ocular discharges, and widespread lymph node enlargement [3,4,10]. However, subclinical infections due to OvGHV2 have been described in several ruminant species, including cattle [20,21] and the American bison [21,22,23]. Pigs infected with OvGHV2 frequently develop the classical clinical manifestations of SA-MCF after contact with the asymptomatic carrier hosts [24,25,26,27,28,29,30], and the acquired disease is similar to that identified in cattle [31]. Surprisingly, pigs reared on commercial farms from Brazil were infected by OvGHV2 and had no contact with sheep [16]. Similarly, there are reports of SA-MCF in horses [32] and cattle [20,33,34,35,36] from diverse geographical biomes of Brazil and in other countries [2,3,37] without direct contact with sheep. It is noteworthy that pigs and other mammalian species are considered the terminal hosts for OvGHV2 [2,3,4,31], and hence do not transmit the virus to susceptible animal populations. Moreover, bison under special conditions, without direct contact with sheep, were infected and developed SA-MCF at a distance of up to 5 km due to aerosol transmission via special wind conditions, mechanical vectors, or even birds [37]. Consequently, one wonders if another animal species may serve as disseminator for OvGHV2.

Free-roaming wild boars (*Sus scrofa*) are predominant within all mesoregions of Paraná State [38]. Recently, our research group investigated the occurrence of OvGHV2 within pulmonary samples of 44 free-ranging wild boars collected from the rural areas of several cities from two distinct mesoregions (Central-northern and Central-eastern) of Paraná State, Southern Brazil [39]. Curiously, OvGHV2 was only identified in wild boars (4.55%; 2/44) from the rural region of the municipality of Castro, in the Central–Eastern mesoregion, where sheep rearing is predominantly extensive on pastures, as opposed to the non-detection in the tissues of wild boars from the Central–Eastern mesoregion where sheep rearing is carried out primarily within enclosed pens [39]. Furthermore, wild boars are recognized carriers for a wide range of bacterial, viral, and parasitic disease pathogens of humans and animals [38,40,41,42], are present as free-roaming animals within all geographical regions and the six biomes of continental Brazil [38], and have been infected by several disease pathogens common to pigs [39]. Therefore, it is plausible to suggest that wild boars may be potential carriers of OvGHV2 and play a role in the dissemination of OvGHV2 to susceptible mammalian populations in the absence of the recognized asymptomatic host. Consequently, this study investigated the occurrence of OvGHV2 in free-ranging wild boars within the Central-northern mesoregion of Paraná State, southern Brazil, a geographical location where sheep are reared predominantly on extensive grass pastures. The main objectives of this study were the following: (1) provide corroborative evidence of OvGHV2-associated infections in free-ranging wild boars; (2) determine the type of infection observed in wild boars by OvGHV2; and (3) evaluate the possible role of wild boars in the epidemiology of OvGHV2-induced infections and/or SA-MCF.

## 2. Materials and Methods

### 2.1. Study Area and Sample Collection

Selected organ fragments (*n* = 13) and biological samples (nasal and oral swabs; *n* = 20) of free-ranging wild boars (*n* = 24) were collected between October 2017 and May 2018 from agricultural and natural areas of Atlantic Forest biome of Paraná State, southern Brazil, including the Vila Velha State Park. Within this conservation unit area, wild boars were baited, photo-monitored, trapped, and euthanized. The in-park trapping was authorized by the Environment Institute of Paraná (authorization number 30/17). These collections sites are within 50 km of the geographical location where OvGHV2 was previously identified within the pulmonary tissues of free-ranging wild boars [39]. Furthermore, all collection sites are located within state-owned conservation units, where the entry of domestic or companion animals is prohibited. The trapping and handling of wild boar samples were approved by the Brazilian Environmental Biodiversity System (SISBIO license 61805–2). This study was also approved by the Ethics Committee of Animal Use (protocol number 059/2017) of the Universidade Federal do Paraná (UFPR).

After collection, organ fragments were immediately placed on ice and transported to the Zoonoses and One Health Laboratory, Department of Veterinary Medicine, UFPR. All biological samples were maintained at −80 °C until used in molecular analyses. Duplicate fragments of organs were immersed in 10% buffered formalin solution for histopathological evaluation.

### 2.2. Histopathological and Immunohistochemical Evaluations

Fragments of the kidneys, liver, lung, and spleen derived from wild boars (#1–14) were immediately immersed in 10% buffered formalin solution. All samples were routinely processed for histopathological evaluation with the Hematoxylin and Eosin stain to identify possible known patterns associated with diseases of domestic animals.

Selected formalin-fixed paraffin-embedded (FFPE) sections of the lungs were used for the IHC detection of intralesional antigens of MCFV using the 15A-MAb, as previously described [11]. Positive controls consisted of FFPE tissue sections known to contain antigens of OvGHV2 from a previous study [43]. The negative control consisted of replacing the 15A-MAb with its diluent, as well as incubating the 15A-MAb on FFPE tissues known to demonstrate negative immunoreactivity to OvGHV2. Negative and positive controls were included in all IHC assays.

### 2.3. DNA Extraction from Organs and Biological Samples of Wild Boars

Nucleic acid extractions from the kidneys, liver, lungs, and spleen were carried out using proteinase K pre-treated aliquots of tissue suspensions from these organs with a combination of the phenol/chloroform/isoamyl alcohol and silica/guanidine isothiocyanate protocols, as described in [44,45]. DNA extraction from the nasal and oral swabs was carried out with the automated extraction system Loccus Extracta 32 (Loccus Equipamentos, São Paulo, Brazil) using the commercial DNA extraction kit (Extracta Kit DNA, Loccus Equipamentos), as recommended by the manufacturer. 

### 2.4. Molecular Detection of OvGHV2 and Specific Viral Pathogens Associated with Porcine Respiratory Disease

The extracted nucleic acids from the organs and swabs were then used in semi-nested PCR (snPCR) assays designed to detect the partial fragment of the OvGHV2 tegument protein gene, as described in [46]. Additionally, the extracted nucleic acids from all pulmonary fragments were used in real-time PCR (qPCR) assays designed to detect influenza A virus (IVA) [47] and porcine circovirus 2 (PCV2) [48]. IVA and PCV2 were included, since these are pulmonary viral pathogens frequently associated with porcine respiratory disease (PRD) in pigs from Brazil [49,50,51]. 

The positive control for the snPCR assay consisted of OvGHV2 DNA derived from a previous study of clinical SA-MCF in cattle [52]. Sterile ultrapure water was used as the negative control in all nucleic acid extractions and subsequent procedures. All PCR products were separated by electrophoresis in 2% agarose gels, stained with ethidium bromide, and examined under ultraviolet light.

### 2.5. Sequencing the Partial Fragment of the OvGHV2 Tegument Protein Gene

The products derived from the semi-nested OvGHV2 PCR assays were purified with a commercial Quick Gel Extraction and PCR Purification Combo kit (Invitrogen^®^ Life Technologies, Carlsbad, CA, USA), quantified using a commercial Fluorometer (Qubit^®^ Invitrogen^®^ Life Technologies, Eugene, OR, USA), and then submitted to direct sequencing in both directions with the forward and reverse primers used in the OvGHV2 nPCR assay in an ABI3500 Genetic Analyzer sequencer. 

Sequence quality analyses and consensus sequences were obtained using PHRED and CAP3 homepage http://asparagin.cenargen.embrapa.br/phph; (accessed 20 March 2024), respectively. BLAST https://blast.ncbi.nlm.nih.gov/Blast.cgi; (accessed 20 March 2024) searches were carried out at GenBank to identify similar nucleotides (nt) of the OvGHV2 tegument protein gene. The nt sequences derived from this study were compared with the prototype strain for OvGHV2 and with strains of OvGHV2 from pigs and/or wild boars.

### 2.6. Types of Macavirus Infections Diagnosed in Free-Ranging Wild Boars from Southern Brazil

The type of *Macavirus*-related infection identified in these wild boars was directedly related to the amplification of OvGHV2 nucleic acids in a wild boar with or without the simultaneous detection of MCFV tissue antigens by IHC [36]. Consequently, wild boars were classified as being infected by OvGHV2 due to the amplification of nucleic acids in any organ evaluated with or without the simultaneous IHC detection of MCFV tissue antigens within any organ. This is because the concomitant IHC identification of MCFV antigens with the amplification of OvGHV2 DNA confirms that the MCFV detected by IHC was indeed OvGHV2. 

Alternatively, infection was considered as MCFV-related due to the detection of tissue antigens without the simultaneous amplification of OvGHV2 DNA. In this case, the detection of MCFV antigens indicated that the related animal was infected by a member of the *Macavirus* genus associated with the development of MCF in their respective hosts. This rational is directly related to the fact that the 15-MAb IHC assay, based on the 15A antigenic epitope [6,7], is detected in all *Macavirus* associated with the development of MCF.

## 3. Results

### 3.1. Principal Histopathological Findings Observed in Free-Ranging Wild Boars

**Figure 1:**
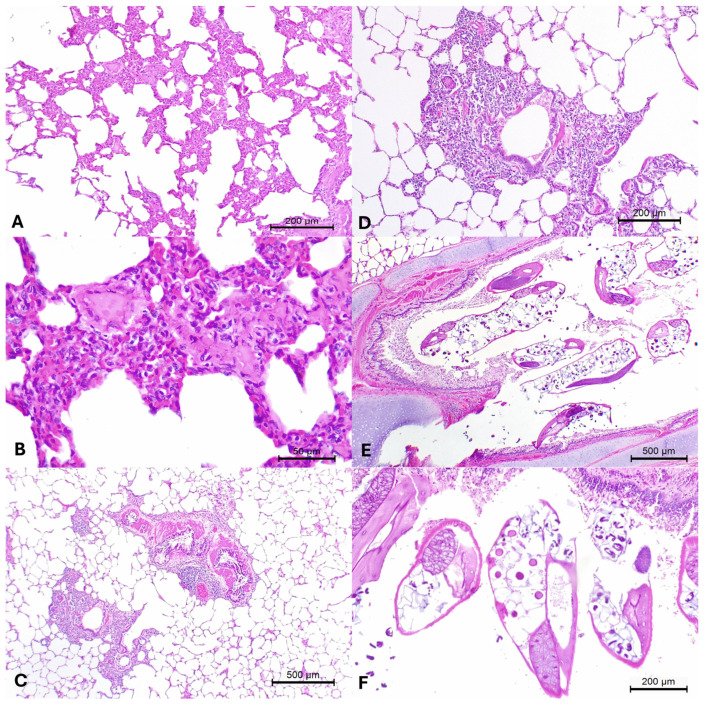
Principal histopathological findings observed in free-ranging wild boars infected with Ovine gammaherpesvirus 2. There is patchy interstitial pneumonia (**A**), which is better appreciated at a closer view (**B**) and bronchointerstitial pneumonia (**C**,**D**). Observe cross-sectional images of intrabronchial nematodes consistent with *Metastrongylus* spp. (**E**,**F**). Hematoxylin and eosin stain. Bars: (**A**,**D**,**F**), 200 µm; (**B**), 50 µm; (**C**,**E**), 500 µm.

The main histopathological patterns associated with the disease of domestic animals observed in the organs of these wild boars are summarized in Table 1. Histopathological evaluations of the lungs identified two distinct patterns of pulmonary disease of domestic animals: interstitial and bronchointerstitial pneumonia [53,54]. Interstitial pneumonia was characterized by mild, patchy areas of thickening of the pulmonary alveolar wall due to the proliferation of type II pneumocytes admixed with lymphoplasmacytic inflammatory cells within the lungs of animals # 11 and 14 (Figure 1A,B). Alternatively, bronchointerstitial pneumonia (Figure 1C,D) was diagnosed due to the marked proliferation of peribronchial histiocytes and lymphocytes [53,54] in wild boars #11 and 13. Additional significant histopathological alterations observed included mild necrohemorrhagic hepatitis with sinusoidal congestion in animals #10 and 11 and parasitic bronchopneumonia (Figure 1E,F) due to the accumulations of pulmonary nematodes consistent with *Metastrongylus* spp. within the large and small bronchi of wild boars # 8, 11, and 13. Additionally, non-specific histological alterations (congestion) were observed within the kidneys and spleens of all wild boars evaluated.

### 3.2. Immunohistochemical Detection of MCFV Antigens in Pulmonary Tissues of Wild Boars

**Figure 2:**
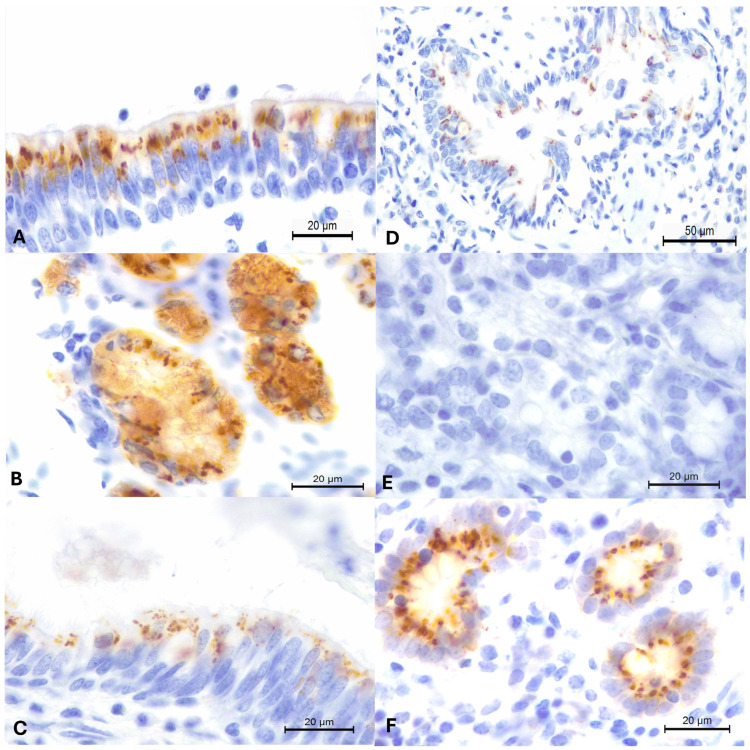
Immunohistochemical detection of malignant catarrhal fever virus (MCFV) antigens in pulmonary tissues of free-ranging wild boars. There is positive intracytoplasmic immunoreactivity to the 15A-MAb within bronchial epithelium (**A**) and epithelial cells of peribronchial glands (**B**) of wild boar #9. Observe positive intracytoplasmic immunoreactivity within the bronchial epithelium (**C**) of wild boar #14 with interstitial pneumonia and within the bronchiolar epithelium (**D**) of wild boar #13 with bronchointerstitial pneumonia. The negative (**E**) and positive (**F**) controls are provided for comparison. Immunoperoxidase counterstained with Hematoxylin. Bars: (**A**–**C**,**E**,**F**), 20 µm; (**D**), 50 µm.

The immunohistochemical findings are presented in Figure 2. There was positive intracytoplasmic immunoreactivity to the 15A-MAb IHC assay with the detection of MCFV tissue antigens within epithelial (bronchiolar and peribronchial glands) cells of the lungs of five wild boars (#2, 9, 10, 13, and 14; Figure 2A,B). Additionally, positive immunoreactivity was observed in sections of the lungs of wild boars with histological evidence of interstitial (#14, Figure 2C) and bronchointerstitial (#10 and 13) pneumonia (Figure 2D), as well as in the normal-looking lungs of wild boars #2 and 9. 

Interestingly, there was positive immunoreactivity to the 15A-MAb in the normal-looking pulmonary tissues of wild boar #2, which contained nucleic acids of OvGHV2. Alternatively, intralesional tissue antigens of MCFV were detected in the lungs of wild boar #14 from the municipality of Palmeira; OvGHV2 nucleic acids were detected within the oral cavity of this animal.

Consequently, two types of subclinical infections were confirmed in these wild boars. The first was related to the molecular detection of nucleic acids of OvGHV2 in nine wild boars (#2, 14, 15, 16, 18, 19, 20, 21, and 24) with and without histological evidence of organ dysfunction. The second was associated with the IHC detection of MCFV tissue antigens within the lungs of three wild boars (#9, 10, and 13).

### 3.3. Molecular Detection of OvGHV2 in Free-Ranging Wild Boars and Phylogenetic Analysis of the Tegument Protein Gene

Frozen tissue sections used in molecular detection were not available for ten wild boars; therefore, only organs from 14 animals were effectively evaluated for the detection of OvGHV2 (Table 1). However, when the results of all samples were analyzed, the snPCR assay with the Baxter primers [46] amplified the 238-base pair (bp) fragment of the OvGHV2 tegument protein gene in 37.5% (9/24) of all evaluated wild boars; direct sequencing confirmed the PCR results of the strains identified in wild boars from the two geographical locations. Selected nt sequences derived from wild boars #11 (trapped at Ponta Grosa) and #14 (trapped at Palmeira) are deposited in GenBank (accession # PP716088 and PP716089, respectively).

Analysis of nt sequences of the OvGHV2 tegument protein gene available in GenBank revealed that there are relative few sequences derived from pigs and wild boars worldwide. The phylogenetic relationship revealed that the OvGHV2 nt sequences derived from the wild boars identified from both geographical locations were identical. Additionally, these two strains (GenBank accession# PP716088 and PP716089) had 99% nt identity with the OvGHV2 reference strain BJ1035 (GenBank accession # NC007646) and 100% nt sequence homology with OvGHV2 strains (GenBank accessions #OQ511653 and OQ511654) identified in two wild boars from the municipality of Castro, southern Brazil [39]. In addition, the strains from this study had 99% nt identity with the strain of OvGHV2 identified in a pig (GenBank accession # HQ223415) with clinical SA-MCF from the state of Minas Gerais, Brazil [16]. 

The distribution of the molecular detection of OvGHV2 DNA from the collected organs and the biological samples of the free-ranging wild boars are summarized (Table 1). OvGHV2 was more frequently (28.7%; 4/14) detected in the lungs, livers, and kidneys as compared with the oral cavity and spleen (7.1%; 1/14). In addition, OvGHV2 was detected in all evaluated tissues from wild boar # 21, the liver and kidney of wild boar #20, and the liver and lungs of wild boar #2. In addition, OvGHV2 was detected in at least one of the evaluated organs from wild boars #15, 18, 19, and 24. Furthermore, OvGHV2 was only detected from the swab derived from the oral cavity of wild boar #14 but was not detected in any of the nasal swabs evaluated. However, when detection was compared only from the tissues and/or biological samples (oral and nasal swabs) effectively collected and analyzed, OvGHV2 was detected in the organs of 57.1% (8/14) of the wild boars and from only 5.9% (1/17) of the biological samples.

Additionally, the qPCR assays did not amplify nucleic acids of IVA and PCV2 from any of the pulmonary samples evaluated in the respective assays. 

### 3.4. Geographical Location of the Collection Sites within the Vila Velha State Park, Paraná State, Southern Brazil

**Figure 3:**
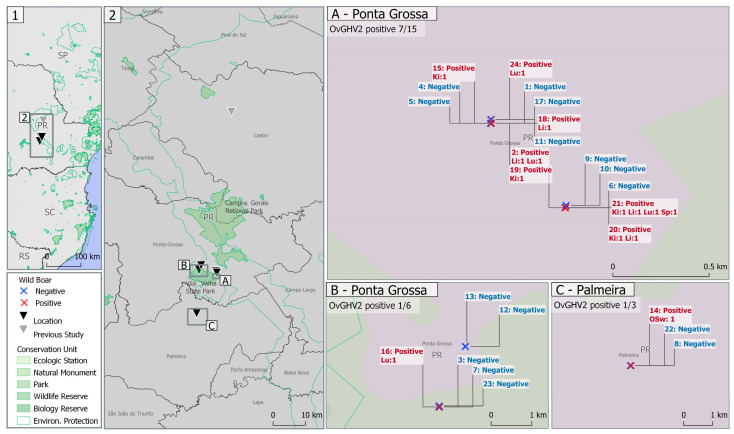
Map of the collection sites within the Vila Velha State Park and agricultural reserves with the distribution of OvGHV2 detected in organs and the oral cavity of free-ranging wild boars from southern Brazil. Lu, lung; Li, liver; Ki, kidney; Sp, spleen; Osw; oral swab.

The geographical locations of the collection sites for all free-ranging wild boars evaluated are illustrated in Figure 3. Detection of OvGHV2 was more elevated in wild boars trapped within the proximity of the Vila Velha State Park (Figure 3.2). Furthermore, OvGHV2 was identified in at least one wild boar from all three regions evaluated, with detection being more elevated within the municipality of Ponta Grossa (Figure 3A,B), relative to Palmeira (Figure 3C). However, molecular detection of OvGHV2 was only identified in the oral swab of one wild boar within the municipality of Palmeira (Figure 3C). 

Additionally, when the molecular detection of OvGHV2 in wild boars from this study was compared with the results of a previous investigation [39], a comparatively more elevated degree of detection was observed within municipalities of the Central-eastern mesoregion as compared the Central-northern and Central-southern mesoregions of Paraná State (Table 2). It must be highlighted that the detection rate of OvGHV2 in free-ranging wild boars within the Central-eastern mesoregion varied between 11.7 and 38.1%, as compared to the non-detection in free-ranging wild boars within municipalities of the Central-northern and Central-southern mesoregions of Paraná State. 

## 4. Discussion

The molecular detection of OvGHV2 DNA from tissues of asymptomatic free-ranging wild boars from this study demonstrated that these animals were probably infected by this virus, considering that the identification of a disease pathogen in organs without any associated lesion is indicative of infection [55]. Moreover, the observation of typical and consistent histological alterations is evidence to support the associated disease agent [56]. Therefore, the identification of interstitial pneumonia in wild boar #14, which contained OvGHV2 DNA within the lungs, suggests that this animal suffered from pulmonary disease due to this viral infection [55,56]; interstitial pneumonia in animals is frequently associated with viral-related infections [53]. Consequently, these findings represent the second report worldwide to relate OvGHV2 with infections in wild boars, since we have previously diagnosed this association in a reduced number of animals [39]. Additionally, the detection of MCFV tissue antigens in three wild boars without the concomitant detection of OVGHV2 DNA may suggest that these animals were infected by another *Macavirus.* However, caution must be taken with the interpretation of this specific finding, since frozen tissues from these wild boars were not available for molecular testing. Therefore, since OvGHV2-related infections are endemic throughout all geographical regions of Brazil [4], it is rather likely that the MCFV antigens detected were those of OvGHV2. During this study, the histological analysis of the organs evaluated from most free-ranging wild boars did not reveal significant pathological patterns consistent with viral- or bacterial-related disease pathogens. However, the lungs of three of these were infested by examples of the lungworm *Metastrongylus* spp.

### 4.1. Free-Ranging Wild Boars Were Probably Infected Due to Incidental Contact with Extensively Reared Asymptomatic Sheep

The identification of additional cases of OvGHV2 in nine free-ranging wild boars within the Central-eastern mesoregion of Paraná state was exciting and yet somewhat surprising. This is because the first detection of OvGHV2 in free-ranging wild boars occurring in trapped animals was diagnosed within the adjacent municipality of Castro in 2017 [39], while sampling for the current study occurred between October 2017 and May 2018. A study that evaluated the movement of wild boars estimated that the distance travelled may vary from 76 km (average 1 km/day) during the resting (sedentary) phase to as far as 310 km (average 6 km/day) during the dispersion phase [57]. Alternatively, another investigation that used the continuous-time speed- and distance-estimation method demonstrated that male wild boars can travel for an average of 5.86 km/day, with females traveling 5.13 km/day [58]. Therefore, a safe estimate suggests that wild boars can travel for approximately 5.8 to 6 km/day. It is also possible that intermixing between wild boars from the two studies could have occurred, considering the periods during which each study was realized independently and the distance between collection sites within the Central-eastern mesoregion. Additionally, male wild boars may travel for considerably longer distances relative to their female counterparts [58]. Consequently, the estimation of the distance travelled by wild boars may be fundamental in understanding how these animals became infected by OvGHV2, considering that sheep are not permitted or reared within these conservation units where all samples were collected. 

We theorize that these free-roaming wild boars would have been in incidental contact with asymptomatic sheep during their daily roaming activities. This is because these conservation units are located approximately 25 and 50 km away from the center of the municipalities of Ponta Grossa and Palmeira, respectively, as well as 50 km from the center of Castro, where the previous detection was confirmed (Figure 3). These three municipalities, and consequently the surrounding sheep-farming regions, are within the estimated sedentary and dispersion distances travelled by wild boars [57]. In addition, the Central-eastern mesoregion where these wild boars were infected has the largest sheep population within the State of Paraná [59]. Moreover, the number of sheep and the specific age are of significant epidemiological importance in the dissemination of SA-MCF [2,37]. In addition, sheep rearing within this region is predominantly extensive on grass pastures [39], and there are frequent sightings of wild boar populations within this mesoregion [38]. Therefore, it is reasonable to suggest that these wild boars were more likely infected not within the premises of the conversation units, but due to incidental contact with asymptomatic sheep reared extensively within the rural areas of this mesoregion. Conversely, the non-detection of OvGHV2 in the samples of free-ranging wild boars collected within the Central-northern region of Paraná State could have been associated with the restricted and enclosed rearing of sheep practiced within these areas, which would have made contact between wild boars and sheep more difficult [39], thereby reducing the likelihood of infection. 

### 4.2. Subclinical OvGHV2-Induced Infections May Produce Pulmonary Disease in Wild Boars

The molecular detection of OvGHV2 in the lungs of four wild boars during this study, two of these with histological evidence of pulmonary disease consistent with viral-induced interstitial pneumonia of domestic animals [53,54], is significant and relates well to the known pathogenesis of this viral infectious disease. Our research group had previously detected OvGHV2 within frozen pulmonary sections of two wild boars, but histological evaluations were not possible [39]. Experimental studies have demonstrated that the initial viral replication of OvGHV2 occurs within type II pneumocytes [60,61]. Interestingly, the simultaneous detection of OvGHV2 nucleic acids and MVFV tissue antigens confirmed that this virus was detected within the normal looking pulmonary tissue of at least one wild boar during this investigation. Furthermore, pulmonary disease is frequently diagnosed in ruminants with clinical SA-MCF [3,4]. In addition, elevated OvGHV2 viral load was detected in a spontaneously infected pig with clinical manifestations of pulmonary impairment and histological evidence of pneumonia [30], and in cows with clinical SA-MCF, from three distinct biomes of Brazil [62]. Moreover, a direct relationship was established between the clinical manifestations of pulmonary discomfort and the development of OvGHV2-associated interstitial pneumonia in dairy calves [43] and adult cows [36]. Therefore, since the histological evidence of pulmonary disease observed in these two wild boars was mild and multifocal, typical of OvGHV2-induced pulmonary disease [60], it is rather likely that these alterations were not sufficient to induce clinical manifestations consistent with pulmonary discomfort. Consequently, these nine wild boars herein investigated, as well as the two from a previous report [39], were arguably subclinically infected by OvGHV2. Hence, the pulmonary disease observed in at least two wild boars is typical of clinical SA-MCF or animals with subclinical infections due to OvGHV2, demonstrating that wild boars subclinically infected with OvGHV2 can develop the typical pattern of virus-induced pneumonia. 

Additionally, infectious agents frequently associated with PRD in pigs from Brazil include IVA, PCV2, *Mycoplasmopsis hyopneumoniae* [49,50,51,63], *Pasteurella multocida* [49,51], *Glaesserella parasuis*, formerly known as *Haemophilus parasuis* [49,63], and *Actinobacillus pleuropneumoniae* [63]. The non-detection of IVA and PCV2 using specific qPCR assays for these pathogens suggested that these viral agents were not associated with the development of the interstitial pneumonia observed in the wild boars during this investigation. 

The possible participation of *P. multocida, G. parasuis* and *A. pleuropneumoniae* in the interstitial pneumonia herein described was excluded, since this pattern of pulmonary disease observed during this study is consistent with viral-induced pneumonia [53,54]. Furthermore, the histopathologic pattern of broncointersticial pneumonia identified in three wild boars is consistent with infections due to *M. hyopneumoniae* (see below) in pigs worldwide. Consequently, further investigations were not realized to fully characterize the etiological bacterial agent associated with this pattern of pulmonary disease in pigs.

Although there are other viral pathogens known to be associated with the development of interstitial pneumonia in pigs worldwide, such as suid herpesvirus 1, porcine inclusion body rhinitis, and porcine reproductive and respiratory syndrome virus, these agents have not been detected in studies that evaluated the occurrence of viral infectious pathogens associated with the development of PRD in pig herds from Brazil [49,50,51]. Therefore, these findings suggest that OvGHV2 was probably associated with interstitial pneumonia observed in a few wild boars during this study. 

### 4.3. Oral Lesions Are Not Manifestations Frequently Associated with OvGHV2-Related Infections in Pigs

The identification of OvGHV2 DNA from only the oral cavity of one wild boar was an interesting and intriguing finding. Since all samples were collected by exotic-wildlife agents not familiar with the diagnosis of infectious disease, it is rather likely that the oral cavity of these animals was not evaluated for possible erosions and/or ulcerations, considering that these are frequently diagnosed in cattle with clinical SA-MCF [2,3,4]. Therefore, the occurrence of oral erosions and/or ulcerations cannot be totally excluded. However, since Paraná State has long been free of foot-and-mouth disease, it is plausible to admit that if these lesions were present, they would have been reported. Additionally, a review of all previously reported cases of SA-MCF in pigs worldwide revealed that oral lesions were described only in an outbreak from the UK [27] and in one pig from a Swiss study [30]. Alternatively, similar lesions were not reported in pigs with SA-MCF from Finland [28], Germany [26], Norway [25], USA [24,29], and Brazil [16]. Therefore, it seems that oral lesions may not be frequent clinical manifestations of OvGHV2-related infections in pigs, so the oral detection of OvGHV2 in animal #21 may be a possible source of dissemination for this virus. 

Most herpesvirus are transmitted by contact due to viral production in the skin and/or presence in the sputum [64], which may explain the detection of OvGHV2 in this asymptomatic wild boar. Alternatively, dissemination of OvGHV2 by the recognized reservoir host occurs predominantly due to virus particles within nasal secretions [1,2,14]. However, as wild boars are not the reservoir hosts, mechanical transfer may be the key for these animals to disseminate viral particles, or these animals may be possible bridge hosts for OvGHV2 [65]. Additionally, the molecular detection of viral DNA within the oral cavity coincided with viremia or viral replication within oral tissues and/or the upper respiratory tract [66], suggesting that this wild boar was probably viremic and hence a potential source for the dissemination of viral particles to susceptible animal populations (see below). Moreover, this wild boar had histological evidence of interstitial pneumonia associated with intralesional MCFV tissue antigens, suggesting that this animal was infected by an MCFV, more likely OvGHV2, which is endemic in all geographical regions of Brazil [4]. However, frozen organs were not available for molecular testing to confirm the participation of this *Macavirus* in the development of this pulmonary disease.

### 4.4. Free-Ranging Wild Boars May Be Potential Candidates for the Dissemination of OvGHV2

Clinical manifestations of SA-MCF without any contact with sheep were reported in diverse geographical locations of continental Brazil, including horses from the Minas Gerais state [32], and cattle from the states of Mato Grosso do Sul [35], São Paulo [33,35], and Paraná [20,34]. Curiously, the activity of free-ranging wild boars is intense and has been expanding within all six existing biomes of Brazil [38]. Additionally, cattle from these outbreaks were mostly aged [35], or from dairy breeds [20], and developed histopathological findings typical of chronic OvGHV2-associated infections [20,35]. Consequently, it is possible that another animal may have served as a possible source of infection in the absence of the recognized asymptomatic host. 

The daily distance travelled by free-ranging wild boars [57,58] may have favored possible contact with susceptible cattle. Consequently, wild boars with viable OvGHV2 in the oral cavity and with tissue antigens may be possible disseminators for this pathogen. This is because OvGHV2 can remain viable within humid environments for at least 13 h [31]. Furthermore, wild boars have been indicated as reservoirs for a wide range of infectious disease pathogens of domestic animals and humans [38,40,41,42], and it would not be surprising if these animals can play a possible role in the mechanical dissemination by the transfer of OvGHV2 to susceptible mammalian populations, or serve as potential bridge hosts [65]. The effects of prevailing winds in aerosol transmission and the possibility of birds as possible mechanical vectors for the dissemination of OvGHV2 to bison were previously considered in the absence of sheep [37]. Therefore, it is probable that subclinically infected free-ranging wild boars may have some role in the mechanical dissemination of OvGHV2, particularly in viremic animals [66]. Alternatively, these wild boars are more likely to serve as bridge hosts for the dissemination of this pathogen [65], since in the mechanical dissemination of infectious diseases, the disseminator is not infected. Consequently, there is initial evidence to suggest the possible role of free-ranging wild boars as potential disseminators for OvGHV2.

### 4.5. The Identification of Bronchointerstitial Pneumonia, Parasitic Bronchopneumonia, and Necrotizing Hepatitis in These Wild Boars Is Not Related to OvGHV2 Infections

The bronchointerstitial pneumonia identified during this study is characteristic of *Mycoplasmopsis* spp.-induced infections in pigs [53,54], and is consistent with previous studies done in Brazil [49,50,51]. Investigations of the etiologic agent associated with pulmonary disease in pigs from different geographical regions of Brazil confirmed that bronchointerstitial pneumonia is frequently associated with infections due to *M. hyopneumoniae* [49,50,51,63,67]. Therefore, it was rather likely that these wild boars were infected by *M. hyopneumoniae*. Additionally, since the pattern of pulmonary lesion associated with porcine mycoplasmosis is different from the pattern observed in infections associated with OvGHV2, further diagnostic investigations were not implemented to clarify the etiology of the bronchointerstitial pneumonia.

The cross-section of the nematodes observed within the pulmonary bronchi of three wild boars are characteristic of infestation by the lungworm *Metastrongylus* spp. [53,54]; similar findings were described in wild boars from Brazil [68] and several European countries [69,70]. Infestations by *Metastrongylus* spp. are relatively common in wild boars, but currently are not frequently diagnosed in commercial pigs, despite been previously associated with the occasional transmission of IAV [53,54]. However, there is no known association of this parasitism with the development of infections due to OvGHV2 or clinical SA-MCF. 

Another interesting histopathological finding observed during this study was necrotizing hepatitis in two animals without an associated portal lymphoplasmacytic inflammatory accumulation. This pattern of hepatic assault is not associated with clinical SA-MCF or subclinical infections due to OvGHV2 in mammals [4], since the hepatic pattern observed in experimentally [12,61] and spontaneously [4,20,28] induced infections generally has accumulations of lymphoplasmacytic inflammatory cells. Accordingly, the possible participation of another specific disease pathogen in the development of these hepatic lesions is being investigated. 

### 4.6. Study Restrictions and Future Perspectives

Although these initial results of subclinical infections due to OvGHV2 in wild boars are interesting and encouraging, additional samples from other geographical regions of Brazil are necessary to understand the possible role of wild boars in the dissemination of OvGHV2. Additionally, a larger number of wild boar samples could have provided more consistent results. Furthermore, the sampling and the type of samples collected was not uniform for all wild boars, which prevented a complete molecular evaluation of the organs of at least 10 wild boars. This setback was most significant in sampling done in wild boars within the region of Ponta Grossa, where the largest percentage of infections was detected. Therefore, the detection rate could have been more elevated within this specific geographical region, had samples been collected from all animals. Nevertheless, these obstacles did not undermine the importance of this investigation, in which it was demonstrated that asymptomatic free-ranging wild boars were subclinically infected by OvGHV2 and may develop pulmonary disease typical of this infection. 

Most of the biological swabs received for molecular investigation were dried, since they were not initially maintained in any transport media or saline solution. From our experience in diagnostic molecular biology for the detection of infectious disease pathogens, nasal and oral swabs maintained immersed in saline solution have a better chance of producing high-quality viable total DNA for molecular testing, as compared to dried samples. Consequently, all extraction of total DNA for these biological samples was performed with an automated extraction system to reduce the manipulation effects that could have occurred were these samples subjected to the phenol/chloroform/isoamyl alcohol and silica/guanidine isothiocyanate protocols, which is far more economical for routine diagnostic laboratory investigations. Therefore, nasal and oral samples should always be maintained immersed in saline solution or other media almost immediately after collection, rather than stored dry. However, it must be highlighted that in diluted samples, the concentration of the target agent and the chances of detection may be reduced [66]. Nevertheless, ideal tissue sampling and preservation for shipment and storage until processing during field sampling and collection from free-ranging wild boars by exotic agents will always be challenging. This is because adequate sampling with subsequent collections may be impaired by unforeseeable environmental conditions such as climate, inadequate light, mud, watery and dusty soil, and the interference of aggressive hunting dogs. Additionally, since wild boar sampling was done only by approved licensed exotic wildlife agents and not by scientists directly involved in research activities, the collection of specific target organs was not always possible. For example, evaluation of the rete mirabile carotid and lymph nodes would have been ideal for the identification of OvGHV2 but was impossible in this actual scenario. 

Finally, the utilization of accelerometer sensors [71] and other tracking methods to monitor the activities of free-ranging wild boars within the biomes of Brazil would be of fundamental importance to establish their potential role in the dissemination of OvGHV2 to susceptible cattle populations within regions where the number of sheep is significantly reduced. Thereafter, serological assays can be used to assess the degree of cattle exposure to this pathogen. Interestingly, serological assays to detect OvGHV2 suggest that this agent is circulating in cows maintained on dairy farms from the Central-eastern mesoregion of Paraná State (manuscript in preparation). 

## 5. Conclusions

OvGHV2 was detected in several organs of asymptomatic free-ranging wild boars and from the nasal cavity of one of these. These initial findings suggest that the free-ranging wild boars herein investigated were subclinically infected. Furthermore, the histological evidence of pulmonary injury with the simultaneous detection of OvGHV2 in the lungs of one wild boar suggests that this animal can demonstrate the typical pulmonary lesion associated with this infection. In addition, the simultaneous detection of intracytoplasmic of MCFV tissue antigens within pulmonary epithelial cells and OvGHV2 DNA in the normally looking lungs of one wild boar demonstrates the potential of this animal to possibly maintain this virus. Although additional studies are required to fully understand the role of free-ranging wild boars in the epidemiology of this viral disease, these initial findings suggest a possible role for these animals in the dissemination of OvGHV2 to susceptible mammalian populations, acting as potential bridge hosts for this pathogen.

## Figures and Tables

**Table 1:** Overview of the collection sites, distribution of OvGHV2 DNA detected in organs and biological samples, and detection of MCFV antigens in free-ranging wild boars from Paraná State, southern Brazil.

Wild Boars #	Collection Sites	Principal Histopathological Disease Pattern ^a^	MCFV IHC ^b^	OvGHV2 snPCR						Type of Infection	
				Lung	Liver	Spleen	Kidney	Nasal Swab	Oral Swab	OvGHV2	MCFV
1	Ponta Grossa A			-ve	-ve	-ve	-ve	-ve	-ve	0	0
2		Normal lung	+ve	+ve	+ve	-ve	-ve	-ve	-ve	1	0
4				NC	NC	NC	NC	-ve	-ve	0	0
5				NC	NC	NC	NC	-ve	-ve	0	0
6				-ve	-ve	-ve	-ve	-ve	-ve	0	0
9		Normal lung	+ve	NC	-ve	-ve	-ve	-ve	-ve	0	1
10		Bronchointerstitial pneumonia; necrohemorrhagic hepatitis	+ve	NC	NC	NC	NC	-ve	-ve	0	1
11		Necrohemorrhagic hepatitis; interstitial pneumonia; lungworm		NC	NC	NC	NC	-ve	-ve	0	0
15				-ve	-ve	-ve	+ve	NC	NC	1	0
17				NC	NC	NC	NC	-ve	-ve	0	0
18				-ve	+ve	-ve	-ve	-ve	-ve	1	0
19				-ve	-ve	-ve	+ve	NC	NC	1	0
`20				-ve	+ve	-ve	+ve	-ve	-ve	1	0
21				+ve	+ve	+ve	+ve	NC	NC	1	0
24				+ve	-ve	-ve	-ve	-ve	-ve	1	0
3	Ponta Grossa B			NC	NC	NC	NC	-ve	-ve	0	0
7				NC	NC	NC	NC	-ve	-ve	0	0
12				NC	NC	NC	NC	-ve	-ve	0	0
13		Bronchointerstitial pneumonia; lungworm	+ve	NC	NC	NC	NC	-ve	-ve	0	1
16				+ve	-ve	-ve	-ve	NC	NC	1	0
23				-ve	-ve	-ve	-ve	-ve	-ve	0	0
8	Palmeira	Lungworm		NC	NC	NC	NC	-ve	-ve	0	0
14		Interstitial pneumonia	+ve	NC	NC	NC	NC	-ve	+ve	1	0
22				-ve	-ve	-ve	-ve	-ve	-ve	0	
*n*				4	4	1	4	0	1	9	3

Legend: ^a^, only significant pathological disease patterns are shown and the normal lung in animals that demonstrated positive immunoreactivity to the 15A-MAb IHC assay; +ve, positive; -ve, negative; NC, not collected; ^b^ MCFV IHC, malignant catarrhal fever virus immunohistochemistry at lungs.

**Table 2:** Molecular detection of OvGHV2 in free-ranging wild boars within municipalities of Paraná State, Southern Brazil.

Mesoregions of Paraná State	Municipalities	OvGHV2 in Free-Ranging Wild Boars			Source
		N^o^ Evaluated	N^o^ Detected	Infected (%)	
Central-eastern	Carambeí	3	0	0	[39]
Central-eastern	Castro	17	2	11.8	[39]
Central-northern	Londrina	14	0	0	[39]
Central-northern	Maringá	2	0	0	[39]
Central-eastern	Palmeira	3	1	33.3	This study
Central-eastern	Ponta Grossa	21	8	38.1	This study
Central-southern	Teixeira Soares	6	0	0	[39]

## Data Availability

The partial nucleotide sequences identified in wild boars during this study are deposited in GenBank (accession # PP716088 and PP716089).
